# Water Skating Miniature Robot Propelled by Acoustic Bubbles

**DOI:** 10.3390/mi14050999

**Published:** 2023-05-04

**Authors:** Hyeonseok Song, Daegeun Kim, Sangkug Chung

**Affiliations:** 1 Department of Mechanical Engineering, Ulsan National Institute of Science and Technology, Ulsan 44919, Republic of Korea; 2 Microsystems, Inc., Yongin 17058, Republic of Korea; 3 Department of Mechanical Engineering, Myongji University, Yongin 17058, Republic of Korea

**Keywords:** cavitational microstreaming, micropropulsion, environment monitoring

## Abstract

This paper presents a miniature robot designed for monitoring its surroundings and exploring small and complex environments by skating on the surface of water. The robot is mainly made of extruded polystyrene insulation (XPS) and Teflon tubes and is propelled by acoustic bubble-induced microstreaming flows generated by gaseous bubbles trapped in the Teflon tubes. The robot’s linear motion, velocity, and rotational motion are tested and measured at different frequencies and voltages. The results show that the propulsion velocity is proportional to the applied voltage but highly depends on the applied frequency. The maximum velocity occurs between the resonant frequencies for two bubbles trapped in Teflon tubes of different lengths. The robot’s maneuvering capability is demonstrated by selective bubble excitation based on the concept of different resonant frequencies for bubbles of different volumes. The proposed water skating robot can perform linear propulsion, rotation, and 2D navigation on the water surface, making it suitable for exploring small and complex water environments.

## 1. Introduction

Recently, interest in research on microrobots has increased with the advanced miniaturization technology of sensors and actuators [1,2]. Accordingly, the development of microrobots that can be used in biomedical and other industrial fields is being carried out [3,4]. These microrobots can be used for not only biomedical microsurgery and targeted drug delivery [5,6] but also for the exploration of small and complex environments [7,8].

The most important issue in the development of microrobots is micropropulsion [9,10]. The micropropulsion method should be different from general macroscale robots because of the low Reynolds number, which is a dimensionless number representing the ratio of the viscous force to the inertial force in each environment [11]. The propulsion method based on the movement of mechanical parts, such as propellers and motors, is not effective in the low Reynolds number environment at the macroscale, where the viscous force is dominant compared to the inertial force [12,13]. Hence, new creative micropropulsion methods at the microscale have been studied.

One of the micropropulsion methods is based on mimicking the motion of tiny living insects and animals. Biomimetic propulsion often utilizes mechanical motion, Marangoni effects, and capillary forces [14]. The propulsion that is induced by mechanical rowing and the walking motion was inspired by the movement of water striders on the water surface. Hu et al. investigated the locomotion of small water insects by high-speed images and flow visualization [15]. Additionally, some research groups developed artificial water striders floating on the water surface using hydrophobic coated legs and demonstrated their mechanical-motion-induced propulsion using electric motors [16,17].

On the other hand, the Marangoni propulsion generated by the surfactant-induced surface tension gradient was inspired by the movement of small aquatic insects, such as Microvelia and Velia [18]. Burton et al. investigated the Marangoni propulsion mechanism, developed a cocktail boat with mimicking the movement of Microvelia and Velia, and demonstrated the boat propulsion on the water surface [19,20].

Capillary propulsion induced by the modification of the surface material property and shape was inspired by the movement of leaf beetle larvae. Hu et al. analyzed the locomotion of meniscus-climbing insects [21]. Additionally, Yu et al. conducted capillary propulsion using a hydrophobic wall and a bent copper sheet [22]. Later, Chung et al. developed an electrowetting on dielectric (EWOD)-driven miniature boat capable of propelling and steering [23,24]. Yuan et al. demonstrated the manipulation of water-floating objects by controlling the surface wettability, and thus, the capillary interaction with EWOD actuation [25]. However, the EWOD-driven capillary propulsion requires wiring connection to supply electric power. Although wireless power transmission was tested, the working distance between the transmitter and the receiver was limited to a few millimeters [26,27]. As alternatives, a new wireless propulsion approach based on cavitational microstreaming flows generated from acoustically excited bubbles has been proposed.

**Figure 1:**
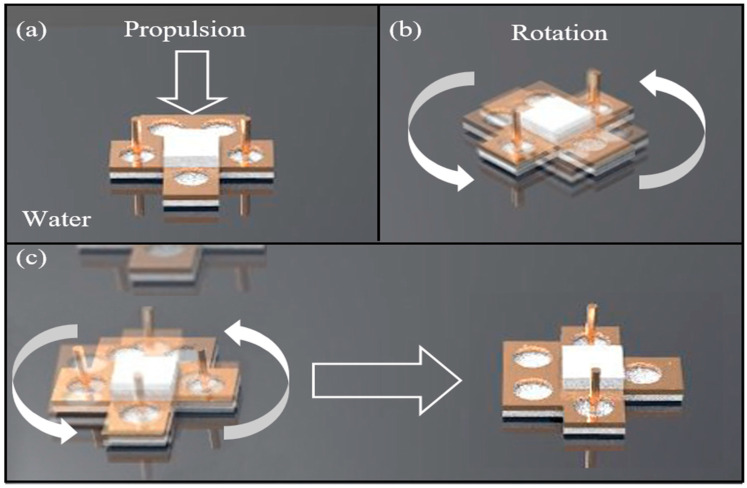
Schematic diagram of the 2D manipulation of a water walking robot actuated by acoustically oscillating bubbles: (**a**) Linear motion; (**b**) Rotational motion; and (**c**) 2D navigation.

**Figure 2:**
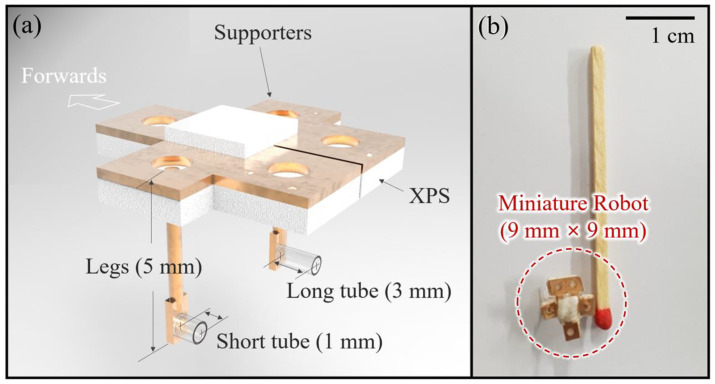
Schematic design and prototype of the proposed water walking robot: (**a**) Schematic water walking robot consisting of extruded polystyrene insulation (XPS), supporters, legs, Teflon tubes; (**b**) Size comparison of the prototype of the water walking robot with a single match.

Dijkink et al. presented a bubble-powered actuator and experimentally demonstrated the rotational operation of the proposed acoustic windmill consisting of tubes partially filled with gas at a speed of a few radians per second [28]. Since then, the bubble-powered actuator has been applied to various miniature robots for their propulsion. In 2011, Won et al. proposed a miniature water-floating boat propelled by acoustic bubbles and demonstrated its linear and rotational motions and two-dimensional (2D) maneuvers [29]. They introduced selective bubble excitation based on the concept of different resonant frequencies for bubbles of different volumes, which enable miniature robots to maneuver in 2D and 3D space. In 2016, Feng et al. showed 2D underwater propulsion using an acoustic bubble-powered microswimmer with microtubes filled with bubbles [30]. Recently, Liu et al. also reported a 3D swimming micro-drone powered by acoustic bubbles using microtubes of different lengths in 2021 [31]. Additionally, Jeong et al. presented an acoustic bubble-powered microrobot for targeted drug delivery based on the same principle of selective bubble excitation [6]. This paper proposes a water skating robot using acoustic bubbles. Figure 1 is a schematic diagram of the proposed water skating robot with a two-dimensional (2D) maneuvering capability. The robot is powered by cavitational microstreaming flows generated from each bubble filled in two Teflon tubes of different lengths and volumes, as seen in Figure 2. When bubbles are acoustically excited by acoustic waves around their resonant frequencies, they oscillate and simultaneously generate microstreaming flows used for propelling the water skating robot, mainly consisting of XPS and Teflon tubes. The XPS with supporters is used for floating the robot on water. Additionally, two Teflon tubes submerged with legs are applied to power the robot based on acoustic-bubble-induced cavitational microstreaming flows. The robot can perform linear propulsion, rotation, and 2D navigation on the water surface.

## 2. Working Principle

When a gaseous bubble is excited by an acoustic wave around the resonant frequency, it oscillates (expands and shrinks) due to its compressibility [32,33]. Additionally, the acoustic bubble generates a quasi-steady cavitational microstreaming flow around it because of the oscillating motion of the bubble–water interface [34]. The acoustically oscillating bubble filled in a miniature tube closed at one end also generates a unidirectional flow and a propulsion force is simultaneously exerted on the tube [35]. The propulsion force (*F*) generated by the acoustically oscillating bubble-induced cavitational microstreaming flow is given as [28]

(1) $$F≃6\rho f^{2}SA^{2}$$

where *ρ* is the liquid density, *f* is the acoustic frequency, *S* is the area of cross section of the microtube, and *A* is the oscillation amplitude of the bubble. The propulsion force is proportional to the acoustic frequency and the bubble oscillation amplitude.

**Figure 3:**
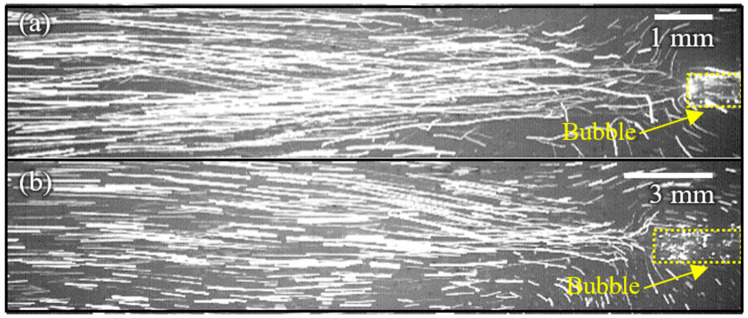
Cavitational microstreaming flows generated from acoustic bubbles trapped in Teflon tubes of different lengths: (**a**) 1 mm long tube; (**b**) 3 mm long tube.

**Figure 4:**
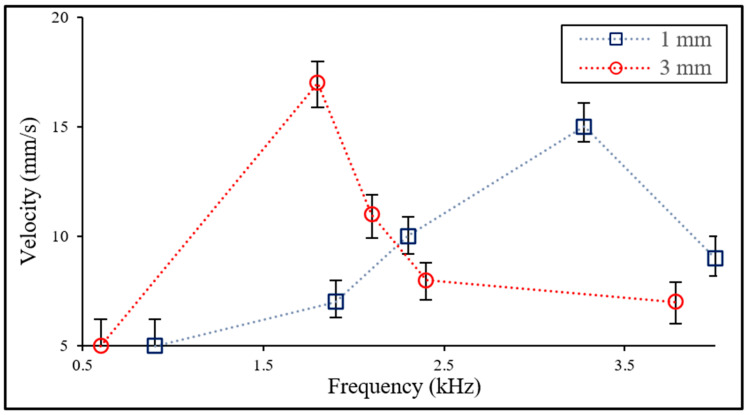
Measurement of the velocity of microstreaming flows generated from acoustically oscillating bubbles filled in Teflon tubes (1 mm and 3 mm lengths) in different frequencies.

The cavitational microstreaming flows generated from each bubble trapped in two Teflon tubes with the same diameter (0.7 mm) and different lengths (1 mm and 3 mm) are visualized using polymer particles (15 μm), as shown in Figure 3. When acoustic waves generated from a disk-shaped piezoactuator (13.5 mm dia.) attach themselves on the side of a water chamber (12.5 (L) × 12.5 (W) × 4 (H) cm), they propagate bubbles in the tubes; the bubbles are acoustically excited and the bubble–water interfaces oscillate in harmony with the applied frequencies. Note that acoustic waves at 3.275 kHz and 1.8 kHz are used for acoustic excitation for each bubble trapped in tubes with 1 mm and 3 mm in length, respectively. The oscillation of the bubble–water interface induces a unidirectional flow with a net momentum used for the propulsion of the proposed water skating robot. The velocity of the flow at 1 mm apart from the inlet is measured in different frequencies using high-speed images, as shown in Figure 4. The result shows that the maximum velocity occurs at each bubble’s resonant frequency. The maximum velocity (17 mm/s) of a 3 mm long tube is about 12% larger than the one of a 1 mm long tube. The measured velocity of each acoustic-bubble-induced cavitational microstreaming flow is used for controlling the linear and rotational motion of the water skating robot.

The experimental setups mainly consist of electrical and optical systems. As a volt-age source for the operation of a piezoactuator (KPR-3020-450, Daeyoung electric, Co., Ltd., Seoul, Republic of Korea), a sinusoidal voltage is generated by a function generator (33210A, Agilent Technologies, Santa Clara, CA, USA) and amplified by a voltage amplifier (PZD700, Trek Inc., New York, NY, USA). All experimental images are captured by a charge-coupled device camera (EO-1312C, Edmund Optics, Barrington, NJ, USA) and a high-speed camera (Phantom Miro eX4, Vision Research, Wayne, NJ, USA) integrated with a zoom lens (VZMTM 450i eo, Edmund Optics, Barrington, NJ, USA) and saved on a personal computer. Particle image velocimetry (PIV) is used for measuring the instantaneous velocity of the tracer polymer particles seeded in the surrounding medium to obtain the flow velocity, along with commercial software (Insight 4GTM, ver.11.0.1.0, TSI Inc., Shoreview, MN, USA).

## 3. Experiment Results and Discussions

**Figure 5:**
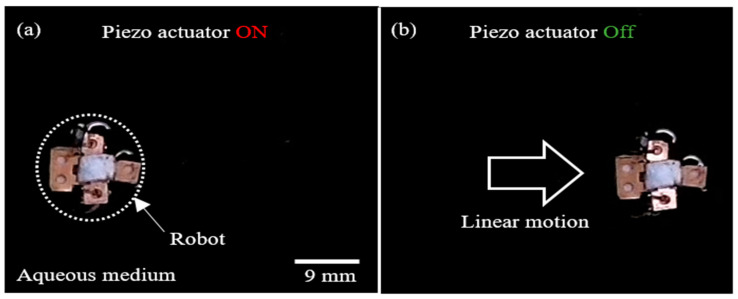
Sequential snapshots of the linear motion of a water walking robot: (**a**) When a piezoactuator is turned on (2.235 kHz), both bubbles trapped in tubes with 1 mm and 3 mm in length generate microstreaming flows, resulting in the robot being linearly propelled in the opposite direction from the flows; (**b**) When the piezoactuator is turned off, the linear motion of the robot stops. Note that the acc wave is applied for approximately 7 s for the motion. Video S1 in the Supplementary Material shows the linear motion of the robot.

The propulsion of the proposed acoustic-bubble-powered water skating robot on the water surface is investigated, as shown in Figure 5. When a piezoactuator (13.5 mm dia.) acoustically excites gaseous bubbles trapped inside two submerged Teflon tubes with the same diameter (0.7 mm) and different lengths (1 mm and 3 mm) at 2.235 kHz, both bubbles simultaneously respond to the applied wave and generate acoustic-bubble-induced microstreaming flows. As a result, the robot propelled in the opposite direction from the microstreaming flows linearly moves to the right by about 20 mm, as shown in Figure 5. However, the linear motion of the robot stops immediately when the piezoactuator is turned off. It confirms that the robot is powered by the acoustic-bubble-induced microstreaming flow.

**Figure 6:**
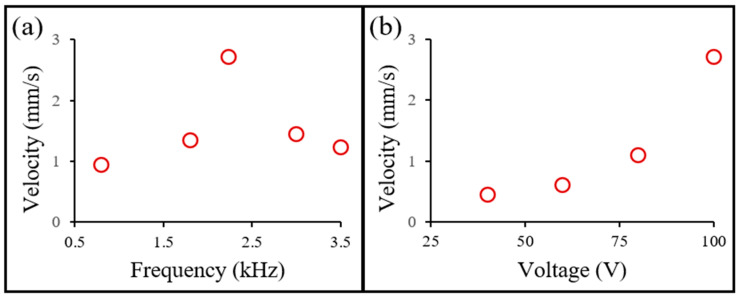
Measurement of the linear motion velocity of a water walking robot actuated by acoustic-bubble-induced microstreaming flows: (**a**) Motion velocities in different frequencies at 100 V; (**b**) Motion velocities in different voltages at 2.235 kHz.

The linear motion velocity of the water skating robot is measured for different frequencies and voltages, as shown in Figure 6. For the frequency, the maximum velocity occurs at 2.235 kHz; that is, between the resonant frequencies for two bubbles trapped in Teflon tubes of different lengths. For the voltage, the motion velocity is proportional to the applied voltage at a fixed frequency (2.235 kHz) with a Pearson correlation coefficient (Benesty et al., 2009) of 0.91 [36].

**Figure 7:**
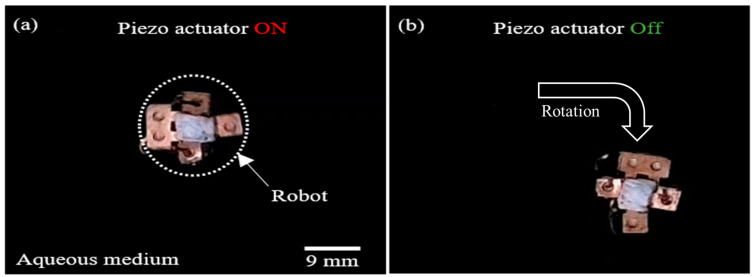
Sequential snapshots of rotational motion of a water walking robot: (**a**) When the piezo actuator is turned on (3.275 kHz), a bubble trapped in a 1 mm tube generates a microstreaming flow, resulting in the robot being rotated in a clockwise direction; (**b**) When the piezo actuator is turned off, the robot stops rotating. Video S2 in the Supplementary Material shows the rotation of the robot.

The rotational motion of the water skating robot is realized by selective bubble excitation based on the concept of different resonant frequencies for bubbles of different volumes. When an acoustic wave (3.275 kHz) generated by the piezoactuator propagates to the bubbles trapped in Teflon tubes of different lengths (1 mm and 3 mm), the bubble trapped in the 1 mm long tube actively respond to the wave and generate the microstreaming flow. On the other hand, the bubble trapped in the 3 mm long tube does not respond to the wave and stays calm because the frequency of the acoustic wave is far away from the resonant frequency of the bubble. Hence, the microstreaming flow from the 3 mm long tube is much stronger than the flow from the 1 mm long tube. As a result, the water skating robot can be rotated in a clockwise direction, as shown in Figure 7.

**Figure 8:**
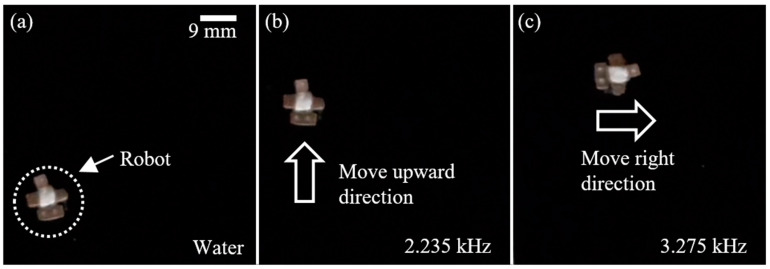
Sequential snapshots of 2D manipulation of the proposed water walking robot: (**a**) Initial state; (**b**) Linear motion at 2.235 kHz; (**c**) Rotational motion at 3.275 kHz.

As proof of concept, the maneuvering capability of the proposed water skating robot is demonstrated. The robot is initially located in the lower left corner, as shown in Figure 8a. When the piezoactuator acoustically excites the bubbles trapped in Teflon tubes at 2.235 kHz, the microstreaming flows generated from both bubbles linearly propel the robot in an upward direction, as shown in Figure 8b. When the applied frequency of the wave is changed from 2.235 kHz to 3.275 kHz, the microstreaming flow from the bubble trapped in the 1 mm long tube is still strong enough to propel the robot. However, the microstreaming flow from the bubble trapped in the 3 mm long tube becomes weak. Hence, the robot rotates in a clockwise direction, as shown in Figure 8c. It shows the high 2D maneuvering capability of the proposed water skating robot.

## 4. Conclusions

This paper presents a water skating robot powered by acoustic-bubble-induced microstreaming flows. First, microstreaming flows generated from acoustically excited bubbles trapped in Teflon tubes with the same diameter (0.7 mm) and different lengths (1 mm and 3 mm) were visualized using polymer particles (15 μm) and measured in different frequencies. The maximum velocity occurs at each bubble’s resonant frequency, and the maximum velocity (20 mm/s) of a 3 mm long tube is about 33% larger than the one of a 1 mm long tube. Second, the propulsion of the robot powered by acoustic-bubble-induced microstreaming flows is conducted, and the propulsion velocities are measured in different frequencies and voltages. For the frequency, the maximum velocity occurs at 2.235 kHz, that is between the resonant frequencies of two bubbles trapped in Teflon tubes of different lengths. For the voltage, the motion velocity is proportional to the applied voltage at a fixed frequency (2.235 kHz). Third, the rotational motion of the water skating robot is also realized by selective bubble excitation. Lastly, the maneuvering capability of the proposed water skating robot is demonstrated, along with performing linear propulsion, rotation, and 2D navigation on the water surface. The proposed water skating miniature robot may be used for applications, such as cell manipulation, drug delivery, microsurgery, and environment monitoring systems.

## Data Availability

Not applicable.

## Supplementary Materials

The following supporting information can be downloaded at: https://www.mdpi.com/article/10.3390/mi14050999/s1, Video S1: Linear propulsion; Video S2: Rotation.
